# A situational analysis of pharmacovigilance plans in the Global Fund Malaria and U.S. President's Malaria Initiative proposals

**DOI:** 10.1186/1475-2875-9-148

**Published:** 2010-05-30

**Authors:** Andy Stergachis, Rebecca JK Bartlein, Alexander Dodoo, Jude Nwokike, S Patrick Kachur

**Affiliations:** 1 Department of Global Health, School of Public Health, Box 357236 University of Washington, Seattle, WA 98195, USA; 2 Department of Epidemiology, School of Public Health, Box 357236 University of Washington, Seattle, WA 98195, USA; 3 Centre for Tropical Clinical Pharmacology & Therapeutics, University of Ghana Medical School, Korle-Bu Teaching Hospital, Accra, Ghana; 4 Strengthening Pharmaceutical Systems, Center for Pharmaceutical Management, Management Sciences for Health, 4301 N. Fairfax Drive, Suite 400, Arlington, VA 22203, USA; 5 Malaria Branch, Division of Parasitic Diseases, Centers for Disease Control and Prevention, Atlanta, GA 30341, USA

## Abstract

**Background:**

Pharmacovigilance programmes can monitor and help ensure the safe use of medicines that are critical to the success of global public health programmes. The widespread deployment of artemisinin-based combination therapy (ACT) by national malaria control programmes as part of the overall Global Malaria Action Plan for malaria control to elimination and eradication makes ACT an excellent candidate for pharmacovigilance activities. In 2008, The Roll Back Malaria partnership issued guidelines for inclusion of pharmacovigilance in Global Fund and other related proposals. In light of this recommendation and the rapid scale-up of ACT worldwide, an analysis of Global Fund Round 8 proposals and the President's Malaria Initiative (PMI) 2009 Malaria Operational Plans was conducted to assess if and how pharmacovigilance has been incorporated into countries' national malaria plans and donor budget requests.

**Methods:**

The Global Fund - Malaria Round 8 proposals for the 26 countries and the PMI Malaria Operational Plans (MOPs) for fiscal year 2009 for the 15 countries that were approved and received funding from either the Global Fund - Malaria Round 8 or PMI were accessed through the programme websites. The analysis consisted of conducting word counts and key word in context analyses of each proposal and plan.

**Results:**

Twelve out of 26 (46%) of the Global Fund proposals mentioned that established pharmacovigilance systems were present in their countries. Four of the fifteen PMI MOPs (27%) mentioned that established pharmacovigilance systems were present in their countries. Only seven of the 26 (27%) Global Fund proposals included a request for funding for new or current pharmacovigilance activities. Seven of 15 (47%) MOPs included a request for funding for pharmacovigilance activities.

**Conclusions:**

There were relatively few requests for funding for pharmacovigilance activities, demonstrating a lack of emphasis placed on pharmacovigilance systems in recipient countries. The findings stress the need for more active direction to strengthen active surveillance and passive adverse event reporting systems to augment the issuance of guidance documents.

## Background

Malaria is a major global public health concern with over 250 million cases annually resulting in close to one million deaths, mostly among young children in sub-Saharan Africa [1]. This devastating disease can be prevented and managed through the proper use of anti-malarial medicines, long-lasting insecticide-treated nets, and indoor residual spraying. The evidence-base for each of these strategies has grown in recent years and has led to the adoption of country policies and guidelines intended to avert malaria cases and save lives [2]. Malaria control efforts have been strengthened by increased funding from numerous donor groups and agencies. Among the largest donor organizations in combating malaria are The Global Fund to Fight AIDS, Tuberculosis and Malaria (Global Fund) and the United States' President's Malaria Initiative (PMI). Increases in donor support combined with renewed drug development efforts and successes in scale-up programmes have resulted in greater access to newer treatments for malaria, such as artemisinin-based combination therapy (ACT).

Introducing newer medicines with limited real-world safety data, such as ACT, into poorly funded health care systems combined with large scale-up access programmes make it imperative to monitor their use and safety. Without efforts to assure that accurate and timely safety information is generated and used, significant resources could be wasted, poor quality products could cause harm, and suboptimal use of medicines could adversely affect patient outcomes and fall short of the goal of improved access to quality, efficacious medicines at affordable cost [3-5]. Pharmacovigilance is the science and activities relating to the detection, evaluation, understanding and prevention of adverse reactions to medicines or any other medicine-related problems and is critical for evaluating and characterizing a drug's risk-benefit profile after being released onto the market [6]. Pharmacovigilance is important in the resource-constrained settings because patients may present different susceptibility profiles for adverse events due to genetic, nutritional, co-morbidity, and other differences and many resource-limited countries lack some or all of the World Health Organization's (WHO) basic elements of a pharmacovigilance system [7,8]. The conduct of pharmacovigilance includes passive reporting of adverse drug events, focused active surveillance studies, the use of confirmatory pharmacoepidemiologic studies, and risk mitigation and risk communication strategies [9]. One of the recommended active surveillance approaches to evaluate systematically the postmarketing safety of medicines used during pregnancy is the use of pregnancy exposure registries, a strategy for pharmacovigilance recommended for products, such as ACT, likely to be used during pregnancy [10-13].

Since the greatest burden of malaria falls upon low- and middle-income countries with inadequate pharmacovigilance systems in place, it is imperative that programmes to support malaria case management also include provisions for pharmacovigilance surveillance. In recognition of this need, the Roll Back Malaria partnership (RBM) issued guidance to countries applying for malaria funding in mid-2008 for the conduct and use of pharmacovigilance within donor-supported programmes. RBM issued recommendations and a template that strongly encouraged all countries to include pharmacovigilance in their national malaria plans as well as to budget for a pharmacovigilance component in any Global Fund or other grant proposal for rapid scale-up of use of ACT [14,15]. In light of these recommendations to increase the attention and funding for pharmacovigilance specific to ACT, Global Fund Round 8 proposals that were due in mid-2008 were analysed. In addition, PMI 2009 Malaria Operational Plans that were due prior to October 2008 were examined to assess if and how pharmacovigilance has been incorporated into countries' national malaria plans and requests made of this major donor.

## Methods

The Global Fund - Malaria Round 8 2008 proposals for the 26 countries and the PMI Malaria Operational Plans (MOPs) for fiscal year 2009 for the 15 countries were accessed from the respective programmes' websites in their entirety (Table 1). All of these proposals were approved and received funding from either the Global Fund - Malaria Round 8 or PMI. Word count and Key Word in Context (KWIC) analyses were conducted on all sections that could contain pharmacovigilance information [16]. In order to evaluate how effectively the RBM guidance was incorporated into the Global Fund proposals, they were examined as well as the PMI MOPs for all specific references to pharmacovigilance-related activities, including budget allocations and evidence of integration of pharmacovigilance into national malaria programmes. Specific search terms were: pharmacovigilance, drug safety, artemisinin, ACT, active surveillance, passive surveillance, post-market surveillance, and pregnancy registry. Both structural and thematic coding was conducted [17] by quantifying the pharmacovigilance references and analysing them for consistency in their definitions of aspects of pharmacovigilance systems such as passive surveillance, active surveillance, and pregnancy registries. Additionally, content analysis of proposals was performed by hand and with ATLAS.ti© software [18].

**Table 1:** Countries included in analyses by type of proposal

Global Fund Round 8 Proposal	Country
http://www.theglobalfund.org/grantdocuments/8AFGM_1614_0_full.pdf	Afghanistan

http://www.theglobalfund.org/grantdocuments/8BOLM_1627_0_full.pdf	Bolivia

http://www.theglobalfund.org/grantdocuments/8BRAM_1632_0_full.pdf	Brazil

http://www.theglobalfund.org/grantdocuments/8BURM_1636_0_full.pdf	Burkina Faso

http://www.theglobalfund.org/grantdocuments/8CAFM_1645_0_full.pdf	Central African Republic

http://www.theglobalfund.org/grantdocuments/8COLM_1652_0_full.pdf	Colombia

http://www.theglobalfund.org/grantdocuments/8COMM_1655_0_full.pdf	Comoros

http://www.theglobalfund.org/grantdocuments/8COGM_1660_0_full.pdf	Congo Republic

http://www.theglobalfund.org/grantdocuments/8ZARM_1658_0_full.pdf	DR of Congo

http://www.theglobalfund.org/grantdocuments/8CIVM_1662_0_full.pdf	Côte d'Ivoire

http://www.theglobalfund.org/grantdocuments/8ECUM_1668_0_full.pdf	Ecuador

http://www.theglobalfund.org/grantdocuments/8ETHM_1672_0_full.pdf	Ethiopia

http://www.theglobalfund.org/grantdocuments/8GHNM_1678_0_full.pdf	Ghana

http://www.theglobalfund.org/grantdocuments/8HTIM_1685_0_full.pdf	Haiti

http://www.theglobalfund.org/grantdocuments/8INDM_1694_0_full.pdf	Indonesia

http://www.theglobalfund.org/grantdocuments/8PRKM_1702_0_full.pdf	Korea

http://www.theglobalfund.org/grantdocuments/8KGZM_1705_0_full.pdf	Kyrgyzstan

http://www.theglobalfund.org/grantdocuments/8NGAM_1730_0_full.pdf	Nigeria

http://www.theglobalfund.org/grantdocuments/8PNGM_1735_0_full.pdf	Papua New Guinea

http://www.theglobalfund.org/grantdocuments/8RWNM_1744_0_full.pdf	Rwanda

http://www.theglobalfund.org/grantdocuments/8SRLM_1756_0_full.pdf	Sri Lanka

http://www.theglobalfund.org/grantdocuments/8SWZM_1759_0_full.pdf	Swaziland

http://www.theglobalfund.org/grantdocuments/8TAJM_1763_0_full.pdf	Tajikistan

http://www.theglobalfund.org/grantdocuments/8TNZM_1766_0_full.pdf	Tanzania

http://www.theglobalfund.org/grantdocuments/8UZBM_1774_0_full.pdf	Uzbekistan

http://www.theglobalfund.org/grantdocuments/8ZANM_1782_0_full.pdf	Zanzibar

http://www.theglobalfund.org/grantdocuments/8ZIMM_1785_0_full.pdf	Zimbabwe

**PMI Malaria Operational Plan**	**Country**

http://fightingmalaria.gov/countries/mops/fy09/angola_mop-fy09.pdf	Angola

http://fightingmalaria.gov/countries/mops/fy09/benin_mop-fy09.pdf	Benin

http://fightingmalaria.gov/countries/mops/fy09/ethiopia_mop-fy09.pdf	Ethiopia

http://fightingmalaria.gov/countries/mops/fy09/ghana_mop-fy09.pdf	Ghana

http://fightingmalaria.gov/countries/mops/fy09/kenya_mop-fy09.pdf	Kenya

http://fightingmalaria.gov/countries/mops/fy09/liberia_mop-fy09.pdf	Liberia

http://fightingmalaria.gov/countries/mops/fy09/madagascar_mop-fy09.pdf	Madagascar

http://fightingmalaria.gov/countries/mops/fy09/malawi_mop-fy09.pdf	Malawi

http://fightingmalaria.gov/countries/mops/fy09/mali_mop-fy09.pdf	Mali

http://fightingmalaria.gov/countries/mops/fy09/mozambique_mop-fy09.pdf	Mozambique

http://fightingmalaria.gov/countries/mops/fy09/rwanda_mop-fy09.pdf	Rwanda

http://fightingmalaria.gov/countries/mops/fy09/senegal_mop-fy09.pdf	Senegal

http://fightingmalaria.gov/countries/mops/fy09/tanzania_mop-fy09.pdf	Tanzania

http://fightingmalaria.gov/countries/mops/fy09/uganda_mop-fy09.pdf	Uganda

http://fightingmalaria.gov/countries/mops/fy09/zambia_mop-fy09.pdf	Zambia

## Results

Twelve out of 26 (46%) Global Fund proposals mentioned that established pharmacovigilance systems were present in their countries (Table 2). When prompted by section 4.10.4 in the Global Fund Round 8 proposal form to cite the integration of current pharmacovigilance activities or to cite future plans, only eight out of 26 countries did so (31%). None of the Global Fund proposals mentioned having or planning to develop a pregnancy registry in their country. Four out of fifteen PMI MOPs (27%) mentioned that established pharmacovigilance systems were present in their countries (Table 2). Only one MOP, from Uganda, mentioned having or planning to develop a pregnancy registry. Four countries had both a MOP and a Global Fund proposal allowing for comparisons between proposals: Ghana, Ethiopia, Rwanda, and Tanzania. Table 3 illustrates inconsistencies in the information submitted by these countries for the PMI MOPs for 2009 and GF Round 8 Malaria proposals. Ghana and Ethiopia, for example, mentioned pharmacovigilance in their Global Fund proposals, but not in the PMI MOP. Tanzania referred to using previous PMI funds to strengthen pharmacovigilance systems, but did not describe pharmacovigilance activities in any detail or request funds for pharmacovigilance in either PMI or Global Fund Round 8 proposal.

**Table 2:** Pharmacovigilance mentions in PMI FY 2009 MOPs and in Global Fund Round 8 - Malaria proposals

	*PMI FY 2009 Countries (15 total)*	*No. of responses (% of total)*	*Global Fund Round 8 Countries (26 total)*	*No. of responses (% of total)*
**Country has a Pharmacovigilance Programme mentioned in MOP or Global Fund proposal**				

Yes	KEN, MDG, MWI, SEN,	4 (27%)	BFA, CAF, COL, COM, CIV, ETH, GHA, NGA, PNG, UZB, ZBR, ZWE	12 (46%)

No	AGO, ETH, GHA, LBR	4 (27%)	AFG, BOL, BRA, COG, ECU, COD, HTI, KGZ, RWA, TJK, TZA,	11 (42%)

Initial Phase of Development	BEN, MLI, MOZ, RWA, UGA	5 (33%)		

No mention in proposal	TZA, ZMB	2 (13%)	IDN, KOR, SWZ,	3 (12%)

Total Respondents		15 (100%)		26 (100%)

**A section is dedicated to pharmacovigilance in the MOP or Global Fund proposal**				

Yes	LBR, MDG, MWI, MLI, MOZ, RWA, SEN, UGA	8 (53%)	COL, COM, COG, COD, ETH, GHA, NGA, ZBR	8 (31%)

No	AGO, BEN, ETH, GHA, KEN, TZA, ZMB	7 (47%)	AFG, BOL, BRA, BFA, CAF, CIV, ECU, HTI, IDN, KOR, KGZ, PNG, RWA, SWZ, TJK, TZA, UZB, ZWE	18 (69%)

Total Respondents		15 (100%)		26 (100%)

**A pregnancy registry is mentioned in the MOP or Global Fund proposal**				

Yes	UGA	1 (7%)	None	0 (0%)

No/Not mentioned in proposal	AGO, BEN, ETH, GHA, KEN, LBR, MDG, MWI, MLI, MOZ, RWA, SEN, TZA, ZMB	14 (93%)	All	26 (100%)

Total Respondents		15 (100%)		26 (100%)

**The country requested funds in its MOP or Global Fund proposal for pharmacovigilance activities:**				

Yes	GHA, MDG, MWI, MLI, MOZ, RWA, UGA	7 (47%)	CAF, COL, COG, CIV, NGA, SWZ, ZBR	7 (27%)

No	AGO, BEN, ETH, KEN, LBR, SEN, TZA, ZMB	8 (53%)	AFG, BOL, BRA, BFA, COM, DR of Congo, ECU, ETH, GHA, HTI, IDN, KOR, KGZ, PNG, RWA, TJK, TZA, UZB, ZWE	19 (73%)

Total Respondents		15 (100%)		26 (100%)

**Type of Pharmacovigilance surveillance mentioned in MOP or Global Fund proposal**				

Passive only	LBR, MWI, UGA	3 (20%)	BFA, CAF, COL, COM, COG, CIV, ETH, NGA, PNG, RWA, UZB, ZBR, ZWE	13 (50%)

Active only		0 (0%)		0 (0%)

Both passive and active	MDG, MLI, RWA	3 (20%)	GHA	1 (4%)

None mentioned	AGO, BEN, ETH, GHA, KEN, MOZ, SEN, TZA, ZMB	9 (60%)	AFG, BOL, BRA, COD, ECU, HTI, IDN, KOR, KGZ, SWZ, TJK, TZA	12 (46%)

Total Respondents		15 (100%)		26 (100%)

**A national agency in charge of Pharmacovigilance activities is mentioned in MOP or Global Fund proposal**				

Yes	GHA, KEN, LBR, MDG, MWI, MLI, MOZ, RWA, SEN, UGA, ZMB	11 (73%)	AFG, BOL, BRA, BFA, CAF, COL, COM, COG, CIV, COD, ETH, GHA, HTI, IDN, KOR, KGZ, NGA, PNG, SWZ, TJK, UZB, ZWE	23 (88%)

No/Not mentioned in proposal	AGO, BEN, ETH, TZA	4 (27%)	ECU, RWA, TZA	3 (12%)

Total Respondents		15 (100%)		26 (100%)

Afghanistan, AFG; Liberia, LBR; Angola, AGO; Angola, AGO; Madagascar, MDG; Benin, BEN; Malawi, MWI; Bolivia, BOL; Mali, MLI; Brazil, BRA; Mozambique, MOZ; Burkina Faso, BFA; Nigeria, NGA; Central African Republic, CAF; Papua New Guinea, PNG; Colombia, COL; Republic of Congo, COG; Cote D'Ivoire, CIV; Rwanda, RWA; Democratic Republic of Congo, COD; Senegal, SEN; Ecuador, ECU; Swaziland, SWZ; Ethiopia, ETH; Tajikistan, TJK; Ghana, GHA; Tanzania, TZA; Haiti, HTI; Uganda, UGA; Indonesia, IDN; Union of Comoros, COM; Kenya, KEN; Uzbekistan, UZB; Korea, KOR; Zambia, ZMB; Kyrgyzstan, KGZ; Zanzibar, ZBR

**Table 3:** Comparison of four countries that submitted both GF Round 8 - Malaria and PMI 2009 proposals

	Country							
	**Ghana**		**Ethiopia**		**Rwanda**		**Tanzania**	

	**PMI FY 2009 MOP**	**Global Fund Round 8**	**PMI FY 2009 MOP**	**Global Fund Round 8**	**PMI FY 2009 MOP**	**Global Fund Round 8**	**PMI FY 2009 MOP**	**Global Fund Round 8**

Pharmacovigilance programme identified in country	No	Yes	No	Yes	In initial phase of devel-opment	No	Not ment-ioned	No

Section dedicated to pharmacovigilance	No	Yes	No	Yes	Yes	Yes	No	Yes

Pregnancy registry identified in country	No	No	No	No	No	No	No	No

Funds requested for pharmacovigilance	Yes	No	No	No	Yes	No	No	No

Type of pharmacovigilance surveillance activities mentioned in the proposal	Not ment-ioned	Passive and active	Not ment-ioned	Passive	Passive and active	Passive	Not ment-ioned	Not ment-ioned

Agency identified in charge of pharmacovigilance activities	Yes	Yes	No	Yes	Yes	No	No	No

The frequency and amount of funding requested for pharmacovigilance by countries seeking funding for malaria programmes was also analysed. Seven of the 26 (27%) Global Fund proposals included a request for funding for pharmacovigilance activities and seven of 15 (47%) MOPs included a request for funding for pharmacovigilance activities. Of the seven countries that requested funds for pharmacovigilance in their PMI MOPs for FY 2009, two included pharmacovigilance within a line item with other treatment and training activities without separating out the component costs. Of the proposals that listed pharmacovigilance projects as an independent line item, the amount requested ranged from $50,000 to $700,000 for activities over three years (Table 4). Analysing the amount requested for pharmacovigilance activities in the Global Fund proposals was not possible due to the manner in which the budgets are delineated; costs are grouped by type (human resources, supplies, etc.) rather than by activity.

**Table 4:** Funding requests to PMI 2009 for pharmacovigilance activities

*Country*	*PMI 2009 - amount requested over 3 years*
Ghana	$110,000^1^

Madagascar	$100,000

Malawi	$700,000

Mali	$400,000

Mozambique	$300,000^1^

Rwanda	$112,100

Uganda	$290,000

^1 ^These figures come from budget line items that include, but are not exclusively pharmacovigilance activities.

## Discussion

This study is, to the authors' knowledge, the first published systematic analysis of country-level pharmacovigilance activities and plans as contained in malaria control and prevention proposals submitted to two of the largest international donor groups. The findings show a lack of systematic and consistent inclusion of pharmacovigilance activities and requests for funding support in proposals and in country plans. This lack of consistency in what was defined and reported as pharmacovigilance across proposals demonstrates the need for stronger efforts to advance the role and effectiveness of the pharmacovigilance field to support malaria control and prevention programmes. The analysis also highlights the need for greater attention to pharmacovigilance in future proposals and country plans. Additionally, this analysis can serve as a baseline for assessing effectiveness of strategies to strengthen programmes over time.

This analysis, in light of the guidance from RBM issued in 2008, demonstrates that such a recommendation had been relatively ineffective and that more is needed to promote the inclusion of pharmacovigilance activities in funding proposals. Required inclusion of pharmacovigilance activities in drug procurement proposals and commensurate budget guidance should be explicitly stated in calls for applications. Recently, as part of the first phase of the Affordable Medicine Facility for malaria (AMFm) initiative, the World Health Organization (WHO) and the Medicines for Malaria Venture (MMV) developed a set of guidelines and recommendations for countries on how and what to include in pharmacovigilance sections of their AMFm proposals [19]. This publication is more detailed than the previous recommendation issued by RBM. Representatives from the Global Fund among other global health initiatives focused on malaria participated in the creation of this guidance, so the inclusion of pharmacovigilance activities should be a serious consideration of future Global Fund Technical Review Panels when evaluating proposals, including funding amounts requested. Future assessments will be needed to measure the impact of this guidance, including analyses of how funding has actually strengthened pharmacovigilance programmes. Budget estimation recommendations and links to potential technical assistance partners would make them even more useful and easily applicable. Delineating the operational details necessary for key methodologies such as active surveillance and stimulated spontaneous reporting could help countries, funders, and review panels develop the same vocabulary with which to describe and propose strengthening of pharmacovigilance structures.

Technical assistance to developing countries seeking to create or enhance their pharmacovigilance systems should be a streamlined, coordinated effort, and standardized reporting forms and mechanisms should be used as much as possible. Much of the infrastructure needed to support a functional pharmacovigilance system is the same as that needed for successful health informatics systems and epidemiologic surveillance. For programmes such as PMI, pharmacovigilance activities should be considered for integration into the current monitoring and evaluation plans, and could be used at the same sites that support sentinel health system or vector surveillance. Recognizing that the most effective pharmacovigilance system is one that is embedded within the health system and linked with the other functions of the existing structure, funders and implementing agencies should work collaboratively with other branches of the health system. This analysis serves as a starting point for future studies of pharmacovigilance activity levels globally. The data presented here serve as baseline indicators and should be tracked to measure the impact that recommendations such as those published by RBM and other future efforts to emphasize the importance of pharmacovigilance system strengthening have on their prominence in global health initiatives' agendas.

The lack of consistency in the descriptions of pharmacovigilance activities and proposed activities (see Additional file 1) underscores the need for coordinating funding streams and more specific strategies to assist in the supporting pharmacovigilance in low-income countries. RBM recommended that a national pharmacovigilance programme for anti-malarial drugs should cost between $150,000 and $250,000 USD for start-up with recurrent costs of around $50,000 per year [20]. This amount would vary depending on the size of a country and the state of existing health infrastructure, such as laboratories, which are key components for pharmacovigilance, as well as other health system activities and the specific activities being proposed. Countries applying for Global Fund support can request support for pharmacovigilance activities under Health Systems Strengthening (HSS) Cross-Cutting Interventions, since a strong pharmacovigilance system is an integral part of a functioning national health system. Global health initiatives are now placing a renewed emphasis on HSS in their programming goals, providing an opportunity to include pharmacovigilance activities within this scope.

There were some limitations to this study. This analysis only presents a snapshot of pharmacovigilance plans and activities as reflected in country plans and key funding proposals for malaria control and prevention. As such, countries' existing pharmacovigilance capabilities and activities may not have been fully captured in this assessment. The budgets for pharmacovigilance in the Global Fund Round 8 proposals were not delineated in such a way as to identify how much funding was requested specifically for pharmacovigilance activities, so they cannot be analysed in the same way that the PMI MOPs can. Descriptions of pharmacovigilance activities and requests for pharmacovigilance support submitted to other funding sources were beyond the scope of this analysis. A brief assessment of the Country Operational Plans (COP) for 2009 submitted by countries that received support from the U.S. President's Emergency Plan for AIDS Relief (PEPFAR) found no mention of pharmacovigilance plans or budget requests in the COPs. Also, no information is presented here as to how the countries used the funds for pharmacovigilance activities as this was beyond the scope of this analysis.

In summary, the key finding from the analysis of both PMI MOPs from FY 2009 as well as GF Round 8 proposals was that overall, there is a dearth of requests for funding for pharmacovigilance programmes, despite acknowledgement by many in the global health community that pharmacovigilance is an integral component of any national malaria programme and health system as a whole. Even within the proposals that did include requests for funding pharmacovigilance programmes, the amounts were often less than recommended. These findings demonstrate the lack of emphasis placed on pharmacovigilance system strengthening despite the fact that in 2008 alone, 15.6 million treatment doses for ACT were procured through PMI in nine of its focus countries [21]. Additionally, it is evident from this analysis that the understanding of what defines a pharmacovigilance system and how much it should cost varies greatly between countries and their assistance partners who help prepare requests to their major donors. Both WHO and the Global Fund are developing a standard set of components needed for a basic functioning pharmacovigilance system. One indicator-based pharmacovigilance assessment tool has recently been developed and pilot tested through the Strengthening Pharmaceutical Systems programme [22]. Pharmacovigilance systems should improve when stakeholders collaborate on better defining and agreeing upon performance indicators and inform where important gaps exist, allowing for all concerned to contribute towards improving the safety of medicines. Investing in pharmacovigilance systems should not only benefit malaria control and prevention programmes, it should also ensure that robust surveillance, reporting and laboratory systems are in place for future monitoring of medications of all kinds.

## Conclusions

Results from the analysis of both PMI MOPs from FY 2009 as well as GF Round 8 proposals indicate that the amount of money requested for pharmacovigilance activities in developing countries do not match up with large-scale deployment of novel anti-malarial treatments and global acknowledgement that pharmacovigilance is an integral component of any national malaria programme and health system as a whole. Within the proposals that did include requests for funding pharmacovigilance programmes, it was difficult to tell what specifically the funds would be used for, and thus to know if sufficient amounts were being requested. For example, if a developing pharmacovigilance system would train and use existing staff to implement surveillance activities, the costs would be much lower than if new workers were necessary to implement the monitoring activities. Finally, there was considerable inconsistency in the pharmacovigilance elements described in requests to PMI and the Global Fund. The format of the proposals themselves, especially the layout of the Global Fund budgets make it difficult to know what specifically the funds will be used to do. Explicitly describing how funds will be used for specific pharmacovigilance activities will aid in achieving true transparency and improve the ability to monitor development of systems. All these findings demonstrate the lack of emphasis placed to-date on pharmacovigilance in recipient countries and among their funding and technical assistance partners as well as a lack of specificity about what the funds are being requested for. Enhanced global guidance and technical assistance could address these shortcomings and would support country and donor goals of ensuring access to high quality and safe treatments for malaria and other conditions. The inconsistencies in describing pharmacovigilance activities between the MOPs and GF proposals of countries that had both underscore the importance of providing well-defined expectations, standards, and tools for establishing and evaluating pharmacovigilance systems.

## Abbreviations

ACT: artemisinin-based combination therapy; AMFm: Affordable Medicine Facility for malaria; COP: Country Operational Plan; KWIC: Key Word in Context; MMV: Medicines for Malaria Venture; MOP: Malaria Operational Plan; PEPFAR: U.S. President's Emergency Plan for AIDS Relief; PMI: President's Malaria Initiative; RBM: Roll Back Malaria; WHO: World Health Organization.

## Competing interests

Jude Nwokike works for the Strengthening Pharmaceutical Systems Program, which receives funding from USAID and specifically from the President’s Malaria Initiative.

## Authors' contributions

AS devised the study design and objectives. AS and RJKB performed the data collection and analysis. All authors contributed to the interpretation of findings and drafting of the text and approved the final manuscript. The findings and conclusions presented in this paper are those of the authors. They do not represent the official position of CDC or the US Public Health Service.

## Supplementary Material

Additional file 1 **Description of proposed pharmacovigilance activities as excerpted from PMI MOPs for fiscal year 2009**. Click here for file
