# Supplementary material for: A situational analysis of pharmacovigilance plans in the Global Fund Malaria and U.S. President's Malaria Initiative proposals
Source: Malar J. 2010 May 30;9:148. doi: 10.1186/1475-2875-9-148 (PMC2887883; doi:10.1186/1475-2875-9-148)
Supplement: Additional file 1 — Description of proposed pharmacovigilance activities as excerpted from PMI MOPs for fiscal year 2009. [file 1475-2875-9-148-S1.DOC]

**Additional file 1. Description of proposed pharmacovigilance activities as excerpted from PMI MOPs for fiscal year 2009[[1]](#footnote-2).**

**Ghana**

Assist in development of national pharmacovigilance programme by supporting the ongoing development and implementation of a pharmacovigilance system including surveillance for adverse drug reactions and rapid response to reports and rumours of severe reactions in direct collaboration with the Ghanaian Food and Drug Board. This activity will build directly on similar activities funded in PMI Year 1. Pharmaceutical and supply chain strengthening activities will also include end-use verification and monitoring of availability of key anti-malarial commodities at the facility level. Specifically, this will entail regular supervisory/monitoring visits to a random sampling of health facilities and regional warehouses to detect and trigger further action on the following critical areas: ACT (or other drug) stock outs; expiration dates of ACT at health facilities; leakage; anomalies in ACT use; and verification of quantification/consumption assumptions.

**Madagascar**

Support is requested to strengthen the national systems for drug quality control and pharmacovigilance. PMI will support the Direction de l'Agence de Medicament de Madagascar (DAMM) to strengthen its capacity to perform frequent and rigorous testing of anti-malarials, especially ACT, in order to ensure high quality ACT in health facilities and communities, and to strengthen and expand the national pharmacovigilance system.

**Malawi**

Continue providing technical assistance and training to the Ministry of Health to strengthen pharmaceutical management at the health facility level. This includes training health workers, helping with quantification, and providing assistance on quality control and post market surveillance.

**Mali**

PMI will continue to support pre- and post-market drug quality monitoring by the national quality control laboratory (Laboratoire National de la Santé or LNS) with equipment and technical assistance. Technical assistance to the LNS will also examine quality of insecticides and ITNs. PMI will also support the development and implementation of a pharmacovigilance plan through the Direction de la Pharmacie et du Médicament (DPM). The pharmacovigilance plan will specifically address adverse events reporting during the widespread implementation of the ACT, artesunate/amodiaquine. PMI will also assist in forecasting ACT needs and improving malaria commodity management by the PPM. PMI will also support the LNS to evaluate anti-malarial drug quality, and will help the DPM establish a system for pharmacovigilance.

**Mozambique**

Support implementation of the new malaria treatment policy by providing training and supervision through site visits to continue supporting the implementation of artemether/lumefantrine (AL) at provincial, district, and health facility levels. Resources will also be provided to support the comprehensive training in malaria management, as part of pre-service training of a cadre of community health workers (APEs), who will be jointly supported by PEPFAR and USAID maternal and child health and reproductive health funding. Pharmacovigilance to monitor adverse reactions to AL and other anti-malarials will also be supported.

**Rwanda**

Support the development of a training manual and job aides, as well as training of health facility staff, on pharmacovigilance of anti-malarials and how to complete the adverse event reporting form. In addition, PMI will support the pharmacovigilance unit of the Pharmacy Taskforce to work closely with the National Programme to Fight against Malaria (PNILP), to ensure that their active pharmacovigilance surveillance activities continue and are integrated into the national system. With increasing importance of community case management (CCM), PMI will support the national pharmacovigilance unit to work in close collaboration with all partners and programmes involved in the implementation of CCM to define the role of pharmacovigilance at the community level. In addition, PMI will support the Pharmacy Task Force to explore integrating pharmacovigilance activities in the private sector approach. One CDC technical assistance visit will be supported in this activity.

**Uganda**

PMI will continue to support the National Drug Authority (NDA) in building a robust pharmacovigilance and quality control system for anti-malarial drugs. Support will be provided for training, sensitization, supportive supervision, adverse effects data collection, and analysis. PMI will also help NDA improve its pre-marketing quality control of anti-malarials entering the country, expand its sentinel sites for post-marketing drug surveillance and fraudulent drug programme to better understand the breadth of fraudulent drug issues in Uganda, and further build its capacity to fulfill its regulatory functions. PMI will also monitor drug resistance (efficacy) of anti-malarial drugs: PMI will continue routine biannual drug efficacy testing of first-line, second-line, and potential alternative anti-malarials in two sites across the country to inform policy decisions.

1. With slight editing modifications for grammar. [↑](#footnote-ref-2)
